# Resource recovery from low strength wastewater in a bioelectrochemical desalination process

**DOI:** 10.1002/elsc.201900048

**Published:** 2019-11-25

**Authors:** Savannah Stuart‐Dahl, Edith Martinez‐Guerra, Bahareh Kokabian, Veera Gnaneswar Gude, Renotta Smith, John Brooks

**Affiliations:** ^1^ Department of Civil and Environmental Engineering Mississippi State University Starkville MS USA; ^2^ Engineer Research and Development Center (ERDC) US Army Corps of Engineers Vicksburg MS USA; ^3^ Center for Academic Success Louisiana State University Baton Rouge LA USA; ^4^ US Department of Agriculture – Agricultural Research Service (USDA‐ARS) Starkville MS USA

**Keywords:** microalgae, microbial desalination, net energy, process optimization and sustainability, wastewater

## Abstract

In this research, low strength synthetic wastewaters with chemical oxygen demand less than 300 mg L^−1^ were treated at different concentrations in a bioelectrochemical desalination process. A process optimization model was utilized to study the performance of the photosynthetic bioelectrochemical desalination process. The variables include substrate (chemical oxygen demand) concentration, total dissolved solids, and microalgae biomass concentration in the cathode chamber. Relationships between the chemical oxygen demand concentration, microalgae, and salt concentrations were evaluated. Power densities and potential energy benefits from microalgal biomass growth were discussed. The results from this study demonstrated the reliability and reproducibility of the photosynthetic microbial desalination process performance followed by a response surface methodology optimization. This study also confirms the suitability of bioelectrochemical desalination process for treating low substrate wastewaters such as agricultural wastewaters, anaerobic digester effluents, and septic tank effluents for net energy production and water desalination.

**Abbreviations**
ANOVA: analysis of variance
COD: chemical oxygen demand
MDC: microbial desalination cell
OTU: Operational Taxonomic Unit
PMDC: photosynthetic microbial desalination cell
RDP: Ribosomal Database Project
RSM: response surface methodology
TDS: total dissolved solids

## 1 INTRODUCTION

**Figure 1:**
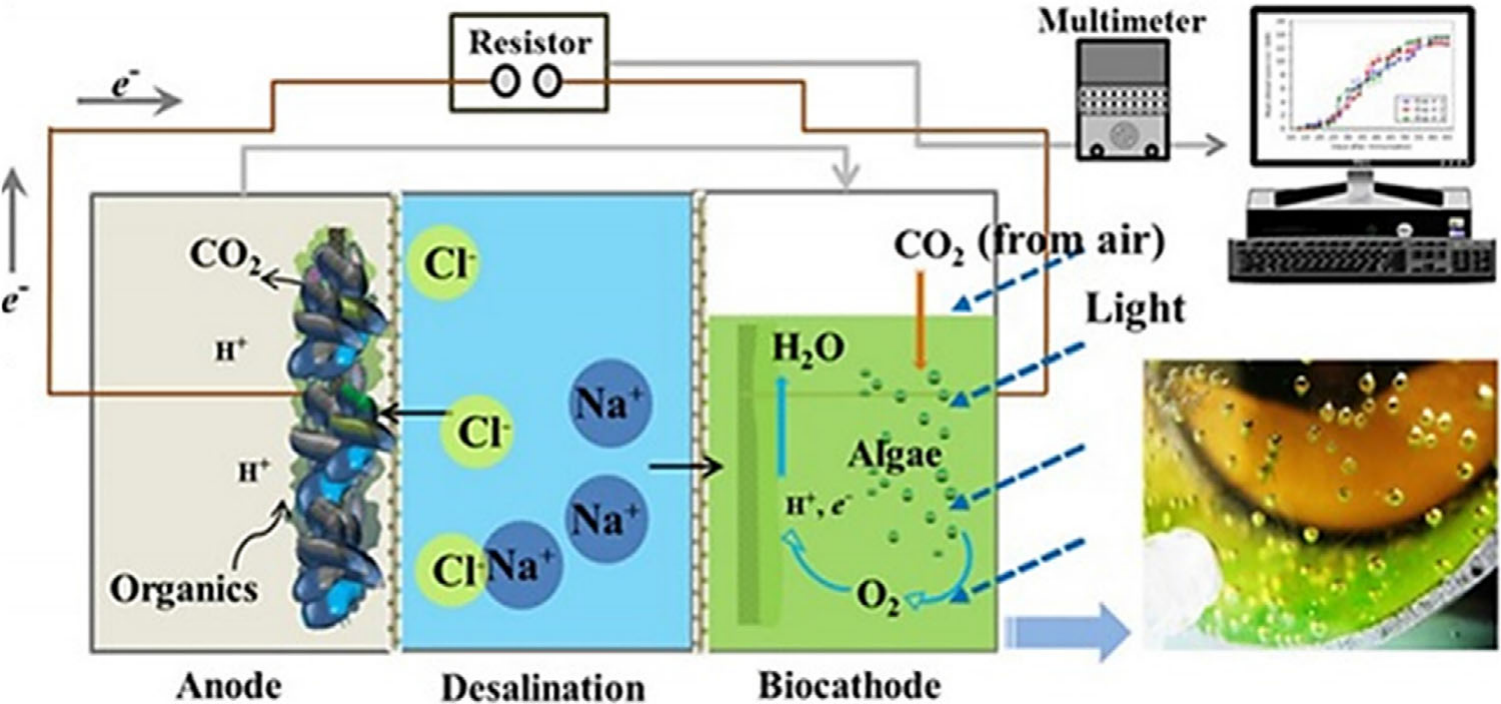
Schematic of the working principle of a photosynthetic bioelectrochemical desalination process (PMDCs) with microalgae biocathode

Groundwater resource management, wastewater reclamation for water reuse, and desalination of saline waters, are the most common alternatives considered when balancing the water supply portfolio of many communities across the world 1, 2, 3, 4, 5. Exploration of new and unconventional water sources (deep saline groundwater and recycled water) caused by groundwater depletion, and consideration of brackish and sea water desalination have become major priorities for many communities 1, 5, 6. Energy and environmental footprints of water and wastewater treatment and desalination processes are well explored in recent literature 4. However, energy and water supply issues are intertwined and cannot be addressed in isolation 6. Integrated solutions that utilize waste sources for energy production, which in turn, power freshwater production are attractive options to address the current energy and water nexus issues 7, 8, 9, 10. To achieve this goal, several biochemical, physico‐chemical, thermochemical, and bioelectrochemical systems have been examined 11, 12, 13. Among these systems, bioelectrochemical systems are of particular interest for their potential and versatility in maximizing the energy and resource recovery from various waste sources. Bioelectrochemical systems can be employed to generate clean electricity or high value energy‐chemical products from various wastewater sources and organic/inorganic wastes that can serve as fuel feedstock for electroactive bacteria and other environmental applications 13, 14, 15, 16, 17, 18. A bioelectrochemical desalination process, which is also known as microbial desalination cells (MDCs), integrate wastewater and saline water treatment for beneficial recovery of energy and other resources but without any external power input or mechanical energy or pressure application 10. To eliminate the environmental issues associated with abiotic anodes and cathodes in bioelectrochemical systems, biological cathodes, especially photosynthetic biocathodes have been developed recently for microbial desalination process, which are known as photosynthetic microbial desalination cells (PMDCs) 10. This system is powered by the biochemical reactions mediated by two different microbial species in bioanode (bacteria) and biocathode (microalgae) compartments 19, 20. The oxidation of organic matter by anaerobic bacteria in the anode chamber results in release of electrons that are transferred through the external electric circuit to the cathode chamber containing microalgae where the reduction process takes place (see Figure 1). Use of photosynthetic bacteria or microalgae in PMDCs can be beneficial in many ways in terms of electron donor supply at anode, organic substrate removal, electron acceptor production, and carbon sequestration by carbon dioxide utilization 21. Figure 1 shows the working principle of a photosynthetic (microalgae) microbial desalination cell. The process details can be found in our previous publications 10.

Although bioelectrochemical systems such as microbial fuel cells, microbial electrolysis cells, and microbial desalination cells, have been studied extensively in recent years, there is still a gap in developing proper process optimization techniques, unified terminology, and methods for the analysis of system performance 22, 23, 24. It is also important to note that though experiments can satisfactorily determine the influence of various factors on the performance characteristics of a bioelectrochemcial system, limitations on the factors of cost and time required to perform the experiments are inevitable. To overcome this limitation, several studies focused on developing mathematical models for estimating the performance of microbial fuel cells and other membrane fuel cells 25, 26, 27, 28, 29, 30, 31, 32, 33.

Studies focusing on long‐term stability and reproducibility of microbial fuel cells are still scarce in literature with the exception of a very few studies 18, 34, 35, 36. Moreover, reproducibility studies and process optimization utilizing low substrate wastewater in microbial desalination cells have not been reported. In this paper, we have evaluated the feasibility of utilizing low substrate wastewaters as an electron donor in microbial desalination cells supported by the microalgae biocathode chamber. The issues related to reliability and reproducibility were addressed through a series of experiments. These were followed by a set of experiments designed using a response surface methodology model and process parametric optimization study of photosynthetic microbial desalination cells for simultaneous energy and water recovery.

**PRACTICAL APPLICATION**

This study elucidated the issues related to reproducibility and reliability of a bioelectrochemically driven desalination process outcomes. It is shown that simultaneous energy and water recovery is possible from low strength wastewaters. However, long term, pilot‐scale studies are required to evaluate the techno‐economic feasibility. This study reported on understanding the interdependence and simultaneous responses of process variables, which is crucial for large scale development.

## 2 MATERIALS AND METHODS

This section will describe the experimental setup, analytical procedures, experimental design details using response surface methodology and applied statistical tools.

### 2.1 Experimental setup

Microbial consortium in the anode compartment was collected from the aerobic sludge of the wastewater treatment plant in Starkville, Mississippi. The sludge was allowed to acclimatize to anaerobic conditions in synthetic wastewater containing 300 mg L^−1^ of chemical oxygen demand (COD) for over 150 days. The microbial consortium was grown in air and microalgae cathode MFCs prior to its transfer into the air and algal MDCs, respectively. The synthetic wastewater in the anode chamber has the following composition (all expressed as “per liter”): glucose 468.7 mg, KH_2_PO_4_ (4.4 g), K_2_HPO_4_ (3.4 g), NH_4_Cl (1.5 g), MgCl_2_ (0.1 g), CaCl_2_ (0.1 g), KCl (0.1 g), MnCl_2_·4H_2_O (0.005 g), and NaMoO_4_·2H_2_O (0.001 g) 21. The COD concentration used in the MDC anode chamber was 500 mg L^−1^. The microalgae *Chlorella vulgaris* used in the cathode compartment was grown in the following mineral solution (all expressed as “per liter”): CaCl_2_ (25 mg), NaCl (25 mg), NaNO_3_ (250 mg), MgSO_4_ (75 mg), KH_2_PO_4_ (105 mg), K_2_HPO_4_ (75 mg), and 3 mL of trace metal solution with the following composition was added to 1000 mL of the above solution (all expressed as “per liter”): FeCl_3_ (0.194 g), MnCl_2_ (0.082 g), CoCl_2_ (0.16 g), Na_2_MoO_4_·2H_2_O (0.008 g), and ZnCl_2_ (0.005 g) 10. *Chlorella vulgaris* sp. was chosen due to its tolerance for high levels of CO_2_ and high efficiency in utilizing CO_2_ through photosynthesis. A known volume of this microalgae consortium with a known cell density was transferred into the cathode chamber. The concentration of microalgae cell biomass was expressed as absorbance (‐). A correlation was established between the absorbance at an optical density of 620 nm and different biomass concentrations (g L^−1^). The relationship between the absorbance (Y) and cell biomass (X) is given as 10:

$$Y=0.8702X\left(R^{2}=0.9962\right)$$

### 2.2 Experimental procedures

The cylindrical‐shaped MFC chambers were made of plexi‐glass and the anode and cathode chambers were separated by an ion exchange membrane. Carbon cloth was used for anode and cathode electrodes. The volume of the anode and cathode chambers was 60 mL after inserting the electrodes. The MDC reactors were prepared by inserting a desalination chamber (30 mL) between anode and cathode chambers in MFC reactors. Cation exchange membrane (CEM, CMI 7000, Membranes international) separated the cathode and desalination part while an anion exchange membrane (AEM, AMI 7001, Membranes international) separated the anode and desalination chambers. The volume of the desalination chamber was about 200 mL with a salt concentration of 10 g L^−1^ NaCl. The volume of the algae chamber was maintained at 100 mL to represent a passive algae biocathode. No external mechanical aeration was provided. Thus, the volume ratios in the photosynthetic MDC system were 2.0:1.0:2.0 for anode, desalination, and cathode chambers, respectively 15.

The voltage was recorded using a digital multi‐meter (Fluke, 287/FVF) and a 10 k ohm resistor was used in closed circuit tests. The current was calculated using the Ohm's law, *I* = *V*/*R*. The power density was calculated (using *P* = *V* × *I*) as per the anode/cathode chamber volume or the electrode surface 37. COD tests were carried out according to the standard methods. Electrical conductivity, total dissolved solids (TDS) removal, and salinity removal were recorded using a conductivity meter (Extech EC400 ExStik Waterproof Conductivity, TDS, Salinity, and Temperature Meter) 37. The pH of the samples was measured using a pH meter (Orion 720A+ advanced ISE/pH/mV/ORP). Dissolved oxygen was measured using a YSI 5100 system. Algae growth was monitored by measuring the optical density of the algal medium with a Spectronic®20 Genesys spectrophotometer at a wavelength of 620 nm. Measurements were taken at regular intervals and three replicates were measured per sample. The desalination rate (*Q*_D_, mg h^−1^) was calculated by *Q*_D_ = (*C*_0_ − *C*_t_)/*t*, where *C*_0_ and *C*_t_ are the initial and the final TDS of saltwater in the middle chamber over a batch cycle of time *t*. Illumination on the microalgae cathode chamber was provided by CFL white light at 60 W (276 µmol m^−2^ s^−1^) 21.

### 2.3 Experimental design using response surface methodology

Response surface methodology (RSM) is a collection of mathematical and statistical analysis tools 38. It has been used in numerous process optimization applications 39, 40, 41, 42, 43, 44. The first step in RSM is to find a suitable approximation to the true relationship. The most common forms are low‐order polynomials (first or second‐order). A second‐order model can significantly improve the optimization process when a first order model suffers lack of fit due to interaction between variables and surface curvature. A general second‐order model is defined as 39:

$$y\;=\beta_{o}\;+\sum_{i\;=\;1}^{k}\beta_{i}X_{i}+\sum_{i<j}^{k}\beta_{ij}X_{i}X_{j}+\sum_{i\;=\;1}^{k}\beta_{ii}\;X_{i}^{2}$$

where Y is the predicted response, *X_i_* and *X_j_* are the input variables that influence the response variable *Y*, *β_0_* is the intercept, *i* represents the linear effect of *X_i_*, *β_ij_* represents the interaction between *X_i_* and *X_j_*, and *β_ii_* represents the quadratic effect of *X_i_*. CCD is one of the modules in RSM to obtain the points of each factor according to their levels 39.

RSM is instrumental in modeling, designing experiments, and establishing the relationship between several independent variables (factors) and the dependent variable 32, 45. RSM helps in predicting the best performance conditions for desirable responses while reducing the number of experimental trials required to evaluate the interaction between multiple factors. A central composite design model with three levels of process variables at three factorial subset design proposed by Gilmour 38 was used to optimize the MDC process in this study. These were represented by a cube with six replications at the center, which offer better approximation of the true error to help determine the significance of process variables 39. The symmetry in design with regard to the center offers equal importance to all levels of all parameters.

The variables (COD, mg L^−1^] and total dissolved solids concentration [g L^−1^], and microalgae concentration [‐]) and levels for the experiment are presented in Table 1A, B, and C are the corresponding values in coded form in Table 1. The criteria for selecting the variables and levels for the experiment are based on our previous experience in this research 10. The bioelectrochemical desalination process performance is influenced by COD and total dissolved solids concentration, and microalgae absorbance in anode, desalination, and biocathode compartments, respectively. A commercial statistical package, Design‐Expert version 7, was used to design experiments and analysis of variance (ANOVA). The process variables were COD (chemical oxygen demand): 100–300 mg L^−1^; TDS (total dissolved solids): 10–30 g L^−1^; and microalgae concentrations measured as absorbance: 0.1–0.3 (dimensionless). The process variable details for the three factorial model are provided in Table 2. ANOVA is a statistical approach that partitions the total variation of a dataset into its component parts for the purpose of testing an assumption on the parameters of the certain selected model. The ANOVA is constructed totally on the basis that the factors are fixed, and the design is crossed. Table 3 depicts the ANOVA for the response surface quadratic model used for voltage production in microbial desalination cells. The quadratic model is a polynomial model containing the linear and two‐factor terms. The sources in the response surface quadratic model include the block, the model, the factors, the residuals, and the lack of fit 45.

**Table 1:** Factors and corresponding coded factors in the RSM study

		Actual levels of coded factors		
Factors	Symbol	−1	0	+1
COD (mg L^−1^)	A	100	200	300
TDS (mg L^−1^)	B	10	20	30
Microalgae absorbance (‐)	C	0.1	0.2	0.3

**Table 2:** Experimental design based on RSM for photosynthetic microbial desalination cell process optimization

Run order	COD (mg L^−1^)	TDS (mg L^−1^)	Microalgae absorbance (−)
1	200	20	0.2
2	100	30	0.3
3	300	10	0.1
4	200	20	0.2
5	100	10	0.3
6	300	30	0.1
7	300	20	0.2
8	200	20	0.2
9	200	10	0.2
10	200	20	0.2
11	100	20	0.2
12	200	30	0.2
13	300	10	0.3
14	200	20	0.1
15	100	10	0.1
16	300	30	0.3
17	200	20	0.2
18	100	30	0.1
19	200	20	0.3
20	200	20	0.2

**Table 3:** ANOVA analysis of voltage production in microbial desalination cells

ANOVA for response surface quadratic model						
ANOVA table [Partial sum of squares–Type III]						
					*p*‐value	
Source	Sum of squares	df	Mean square	F‐value	Prob > F	
Model	6.126E‐003	9	6.806E‐004	3.25	0.0401	Significant
A‐COD	1.464E‐005	1	1.464E‐005	0.070	0.7968	Not significant
B‐TDS	7.465E‐004	1	7.465E‐004	3.57	0.0883	Not significant
C‐Algae absorbance	9.120E‐005	1	9.120E‐005	0.44	0.5241	Not significant
AB	2.168E‐003	1	2.168E‐003	10.36	0.0092	Significant
AC	1.044E‐004	1	1.044E‐004	0.50	0.4962	Not significant
BC	2.365E‐004	1	2.365E‐004	1.13	0.3128	Not significant
A^2^	9.751E‐005	1	9.751E‐005	0.47	0.5104	Not significant
B^2^	2.155E‐003	1	2.155E‐003	10.30	0.0094	Significant
C^2^	2.163E‐005	1	2.163E‐005	0.10	0.7545	Not significant
Residual	2.093E‐003	10	2.093E‐004			
Lack of fit	1.596E‐003	5	3.193E‐004	3.21	0.1131	Not significant
Pure error	4.970E‐004	5	9.940E‐005			
Total	8.219E‐003	19				

### 2.4 Microbial composition evaluation — DNA extraction and PCR amplification

Sample DNA was extracted using a FastDNA Spin Kit for soil (MPbio) in conjunction with a FastPrep FP120 (BIO 101) operated at 40s. Samples were extracted from different locations, including: (1) anode suspension and sediments; (2) electrode biofilm (carbon cloth); (3) anaerobic source; and (4) purple solids formed in the anode chamber. Electrode biofilm carbon electrode was first prepared by aseptically cutting the paper into small pieces using sterilized scissors and pieces placed in a 2 mL centrifuge tube. Tubes were loaded with the first solution from the FastDNA Spin kit and homogenized with the FastPrep FP120. Purple solids attached to the wall of the anode chamber were scrubbed with sterile forceps from the MDC anode chamber's internal wall and placed in a 2 mL microcentrifuge tube. Manufacturer's recommended extraction protocol was followed prior to PCR amplification.

PCR amplification was carried out as stated in each respective reference. Otherwise, samples were amplified in a Hybaid MBS 0.2 G thermal cycler with an initial DNA denaturation for 10 min at 95°C, followed by 30 cycles of 30 s at 95°C, 30 s at 55°C, and 30 s at 72°C, and then final cycle for 10 min at 72°C. After PCR amplification, products were loaded on 0.5 TAE agarose gel, electrophoresed, and visualized on an Alpha Biotech AlphaImager. In addition to the aforementioned PCR assays, qPCR of the 16S rRNA gene was also conducted. Briefly, a 25 µL reaction mixture containing 12.5 µL ABI Syber Green Master mix, 0.5 µL of primers, and 2 µL of template DNA in conjunction with universal 16S rRNA primers as stated in 46.

### 2.5 16S rRNA high throughput sequencing

At the fifth month of consecutive operation, four different samples were collected from different locations of the MDC to determine variations in bacterial communities using high throughput 16S rRNA sequencing. These samples included anode sediments, electrode biofilm C paper, anaerobic source, and purple solids formed in the anode chamber. Soil microbial genomic DNA was submitted for library preparation and sequencing through Global Biologics Molecular Genetics and Sequencing Services (Columbia, MO) and processed for Illumina MiSeq DNA sequencing using 2 × 300 bp paired‐end sequencing. Briefly, the V3V4 region of the 16S rRNA gene was amplified, sequenced, and analyzed. Bioinformatic 16S rRNA sequence analysis was carried out using the Mothur platform (v. 1.39.5) following the Miseq SOP as outlined on the mothur website (http://www.mothur.org/wiki/miseq_sop) 47 and utilizing tools available on the Ribosomal Database Project (RDP) pipeline. Briefly, libraries were contiged and curated to reduce errors and low‐quality sequences using Mothur. Sequences which did not align or classify as *Eubacteria* were removed from the libraries. Chimera sequences were screened within Mothur. Operational Taxonomic Units (OTUs) were assigned at a 3% dissimilarity. OTU and phylotype analyses were conducted using Mothur 47. Briefly, OTU‐based analysis consisted of alpha and beta‐diversity analyses comprised of invsimpson, jclass, thetayc, parsimony, and amova commands. Additionally, sequences were processed utilizing the RDP high throughput sequencing pipeline using RDP‐classifier, aligner, complete linkage clustering, Shannon/Chao1 Index, Jaccard/Sorensen Index, and RDP Lib Compare.

## 3 RESULTS AND DISCUSSION

This section will first describe the studies conducted to evaluate reproducibility of the microbial desalination cells. Once the cells are stabilized, the process optimization study was conducted followed by collection of microbial samples for microbiome analysis from different sections of PMDCs except the biocathode compartment. The results obtained in these experimental studies are presented in the following sections.

### 3.1 Reproducibility in photosynthetic microbial desalination cells

**Figure 2:**
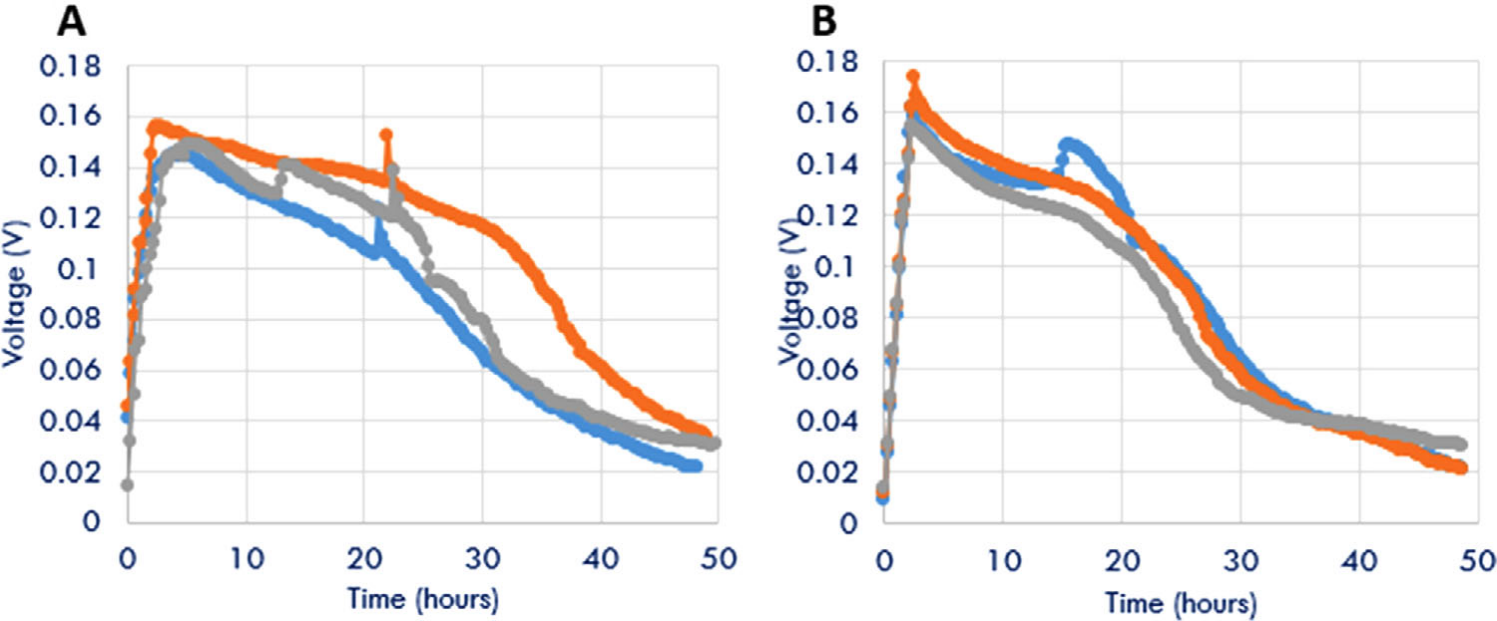
Voltage production profiles for three different MDCs in two sequential tests (total six tests at 200 mg L^−1^ COD, 20 mg L^−1^ TDS, and 0.2 absorbance)

The experimental studies were conducted in two phases in which the first phase evaluated the reproducibility of the outcomes of the microbial desalination cells (Figure 2). Three identical MDC reactors were developed and operated with similar process conditions. Two sets of tests were conducted over 2‐day process time. The process conditions were fixed at 200 mg L^−1^ of COD, 20 g L^−1^ of TDS, and 0.2 microalgae absorbance. Results shown in Figure 2 demonstrate the reproducibility of the MDCs. The voltage production with time and cumulative voltages were in acceptable range for all the tests. For example, a maximum voltage production rate ranging between 0.15 and 0.17 V was observed among the two different tests in the six MDC reactors.

### 3.2 Evaluation of electricity production in photosynthetic microbial desalination cells

**Figure 3:**
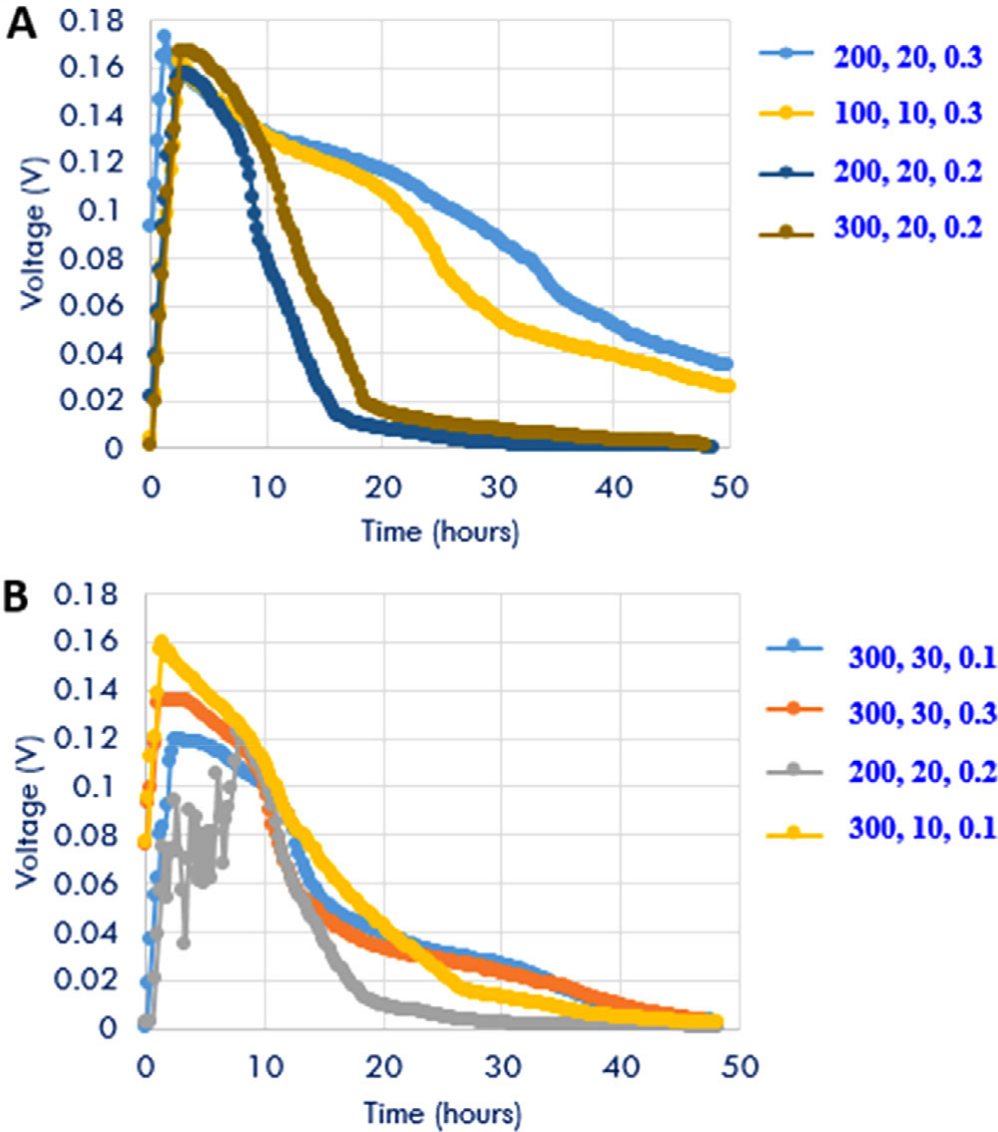
(A and B) Voltage production profiles for eight different test conditions in photosynthetic MDCs

Figure 3 shows the voltage production and cumulative voltage output profiles for eight different test conditions in MDCs using response surface methodology as shown in Table 2. It can be noted that higher voltage was produced for higher COD concentrations due to availability of organic matter over a long period of time. Some tests were repeated to confirm the response of the process parameters where

On the other hand, there are competing biochemical reactions promoted by different microbial populations in bioelectrochemical system, especially in those fed by mixed consortium. Substrates in bioanode chamber are utilized by different populations of microorganism to produce methane (by methanogens) and electricity (exoelectrogens). Substrate concentrations play an important role in the performance of bioelectrochemical systems. For instance, a comparison of competition between the electrogens and methanogens through the experimental studies has proven that low substrate loading in the bioanode will reduce methanogenic activity 17, 29, 48. High specific anode surface area with high overpotential can improve the current density in bioelectrochemical systems. From this perspective, the use of low substrate wastewaters or substrates for bioelectricity production in PMDCs could prove to be beneficial.

### 3.3 Process optimization by response surface methodology tool

#### 3.3.1 Evaluation of response surface methodology model

**Figure 4:**
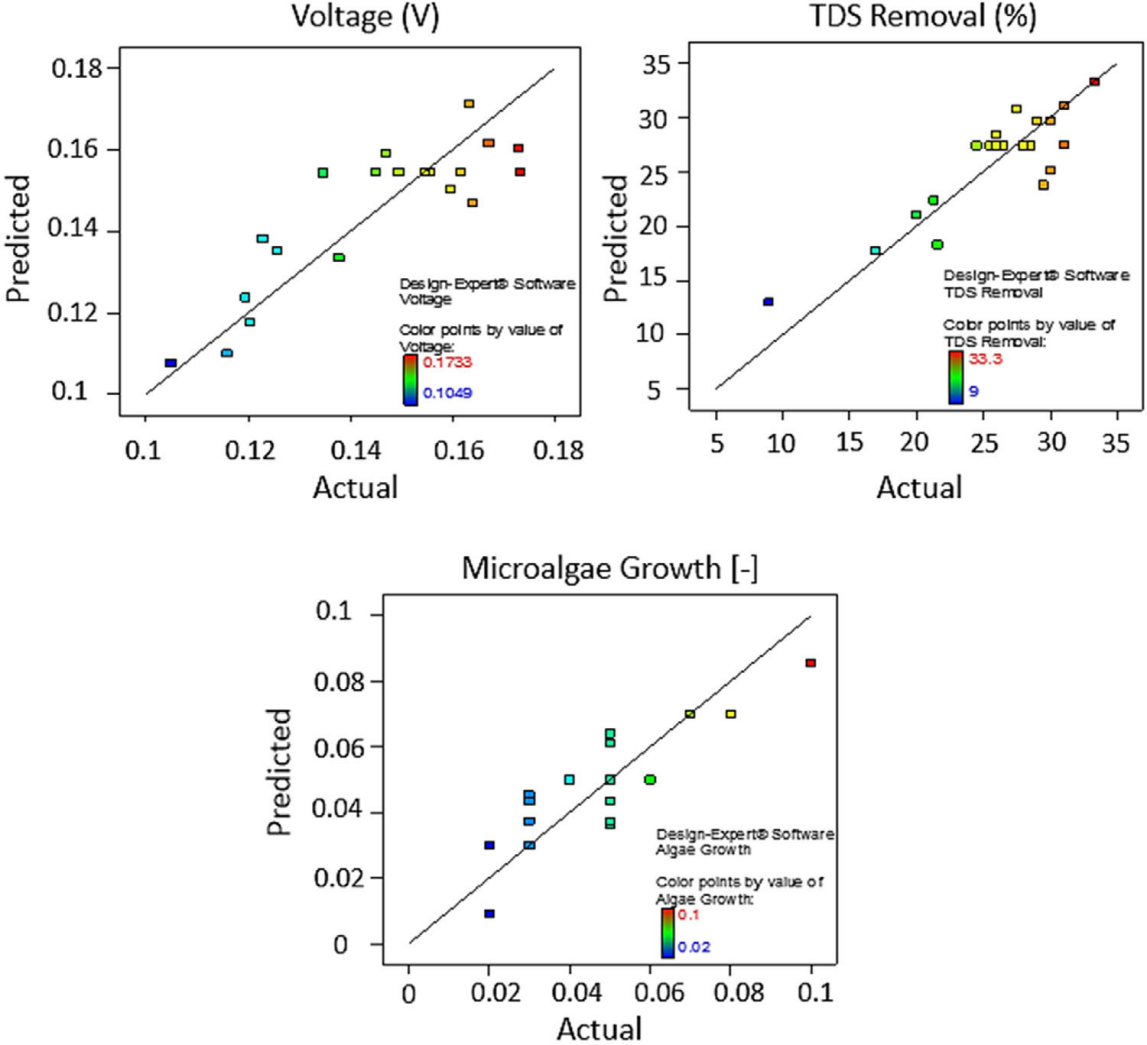
Actual vs. predicted values and the fit for different effects observed in MDCs: voltage production; TDS removal; and microalgae growth

Using CCD, a number of experiments were designed to optimize the operation of MDC and to establish the optimal levels of selected variables (chemical oxygen demand, COD; total dissolved solids, TDS; and microalgae biomass) for maximum conversion of COD into bioelectricity, water desalination, and microalgae biomass growth rates. A total of 20 experiments (excluding the previous reproducibility experiments in Sections 3.1 and 3.2) were performed to understand the effect of these variables. The coded range (high, zero, and low levels) of the factors and the results of the experiments (actual and predicted values) are shown in Figure 4. Mathematical analysis of variables and results are shown in Table 3. The model *p*‐value of <0.05 indicates the higher significance of the corresponding model and the *p*‐value obtained in the present work from the ANOVA table was less than 0.05 confirming that the quadratic model is highly suitable and statistically significant. Results from the ANOVA analysis (Table 3) also indicate that among linear terms (A: COD; B: TDS, and C: microalgae biomass) the effect of COD, TDS, and microalgae biomass was not that significant compared to the interactive effects between COD and TDS concentrations (AB). For example, it is clear that COD concentration can affect the function of MDC by impacting the voltage production and proton transfer between the anode and desalination chambers. In addition, COD removal can be affected by the TDS concentration which prompts ionic transfer in the MDCs. Similarly, TDS removal or ionic transfer in desalination chamber is affected by the exoelectrogenic activity influenced by the availability of COD concentrations. The quadratic effect of TDS removal or concentration (B^2^) was also significant as explained by the previous factors. The lack of fit of this model was 0.1131, which is greater than 0.05 making it non‐significant. Using the RSM analysis, the following relationships were derived for voltage production, TDS removal, and microalgae growth rates in bioanode, desalination, and biocathode chambers.

##### Voltage generation

*Final equation in terms of coded factors*:

Voltage = +1.210E‐003 × A ‐ 8.640E‐003 × B + 3.020E‐003 × C + 0.016 × AB ‐ 3.613E‐003 × AC‐5.437E‐003 × BC + 5.955E‐003 × A^2^ ‐ 0.028 × B^2^ + 2.805E‐003 × C^2^

The equation in terms of coded factors can be used to make predictions about the response for given levels of each factor.

*Final equation in terms of actual factors*:

Voltage = +0.11591 ‐ 4.83082E‐004 × COD +8.12918E‐003 × TDS +0.099018 × Microalgae Absorbance +1.64625E‐005 × COD × TDS −3.61250E‐004 × COD × Microalgae Absorbance −5.43750E‐003 × TDS × Microalgae Absorbance +5.95455E‐007 × COD^2^ −2.79955E‐004 × TDS^2^+0.28045 × Microalgae Absorbance^2^

##### TDS removal

*Final equation in terms of coded factors*:

TDS Removal = +27.34 +1.69 × A +2.54 × B −3.49 × C +3.71 × AB +3.64 × AC −1.21 × BC −0.60 × A^2^ −2.45 × B^2^ −0.10 × C^2^

*Final equation in terms of actual factors*:

TDS Removal = +37.75727 −0.10592 × COD +0.73582 × TDS −79.21818 × Microalgae Absorbance +3.71250E‐003 × COD × TDS +0.36375 × COD × Microalgae Absorbance −1.21250 × TDS × Microalgae Absorbance −6.04545E‐005 × COD^2^ −0.024545 × TDS^2^ −10.45455 × Microalgae Absorbance^2^

##### Microalgae growth

*Final equation in terms of coded factors*:

Algae Growth = +0.050 −3.000E‐003 × A +3.000E‐003 × B −0.020 × C + 3.750E‐003 × AB −3.750E‐003 × AC +3.750E‐003 × BC −9.545E‐003 × A^2^ −9.545E‐003 × B^2^ +0.015 × C^2^

*Final equation in terms of actual factors*:

Microalgae Growth = +0.090273 +3.51818E‐004 × COD +2.61818E‐003 × TDS −0.81818 × Microalgae Absorbance +3.75000E‐006 × COD × TDS −3.75000E‐004 × COD × Microalgae Absorbance +3.75000E‐003 × TDS × Microalgae Absorbance −9.54545E‐007 × COD^2^ −9.54545E‐005 × TDS^2^ +1.54545 × Microalgae Absorbance^2^

The results obtained in this work are similar to some of the previous observations using process optimization techniques 49. In this study, we noticed the interaction and quadratic effects to be significant as reported in other studies focusing on microbial fuel cells 42, 50, 51, 52, 53, 54. It should be noted that the microbial desalination cells are based on the same fundamental bio‐electrochemical principle as in microbial fuel cells. The individual and interactive effects can vary depending on the process configuration, nature, and characteristics of the process streams and operating conditions 55.

#### 3.3.2 Relationship between voltage, COD, TDS removal, and microalgae absorbance

**Figure 5:**
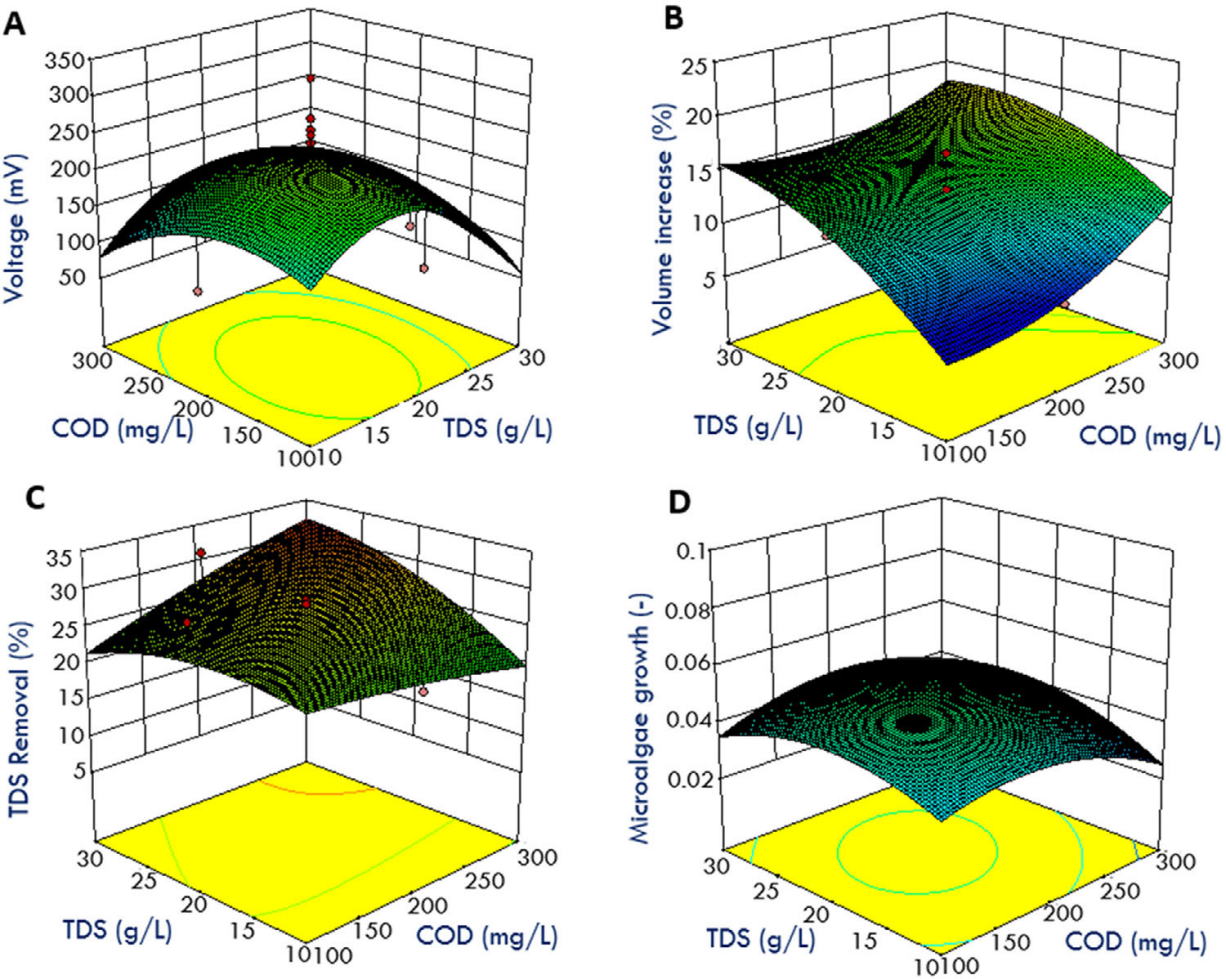
The relationship between (A) the voltage production (mV), (B) volume gain in desalination chamber (%); (C) desalination rate (%) and (D) microalgae growth influenced by the process conditions COD (mg L^−1^); TDS (g L^−1^), and microalgae absorbance (dimensionless) using RSM optimization model

The interdependence of COD (mg L^−1^), TDS (g L^−1^), and microalgae concentration (absorbance) and their effect on the voltage production are depicted in Figure 5A. A voltage production of 200 mV can be achieved at 250 mg/L, TDS of 20 g L^−1^, and microalgae absorbance of 0.2. Higher TDS and COD concentrations did not necessarily increase the voltage output probably due to increased ionic transfer. The relationship between the desalination rate (or the increase in the volume of desalinated water as percentage in the middle chamber) and the TDS (g L^−1^) and algae absorbance are shown in Figure 4C and D. Higher TDS and COD concentrations favored desalination rate as shown in Figure 4C and D. The desalination rate was 18% at a TDS concentration of 25 (g L^−1^) and COD of 300 (mg L^−1^). The relationship between various parameters affecting the salt removal and microalgae growth are shown in Figure 5B. Microalgae biomass concentration did not necessarily have an effect on the volume gain in the desalination chamber as the microalgae concentration does not represent dissolved ionic concentration. As shown in Figure 5B, higher COD and TDS concentrations favor higher TDS removal (desalination rate). However, an inverse relationship can be observed for microalgae absorbance vs. TD removal.

The relationship between various parameters affecting the salt removal and microalgae growth are shown in Figure 5C. Higher concentrations of both COD and TDS in anode and desalination chambers are favoring the TDS removal rate facilitated by higher electron production and therefore, higher movement of chloride from the desalination chamber to the anode chamber (Figure 5C). On the other hand, lower microalgae concentrations are suitable for higher TDS removal because the desalination process between the desalination and biocathode chamber is more dominated by the diffusion process where the concentration difference serves as the driving force whereas between the anode and salt chamber the desalination process is more favored by the electron release and proton transfer process. Relationship between microalgae growth and COD, TDS and microalgae biomass concentrations is shown in Figure 5D. Microalgae growth was higher at COD and TDS concentrations of 200–250 mg L^−1^ and 20–25 g L^−1^, respectively. In addition, microalgae growth rate was higher for lower initial microalgae concentrations and not affected significantly with increasing initial concentrations which could be limited by the availability of nutrients and light conditions.

### 3.4 Energy aspects of bioelectrochemical desalination process

**Figure 6:**
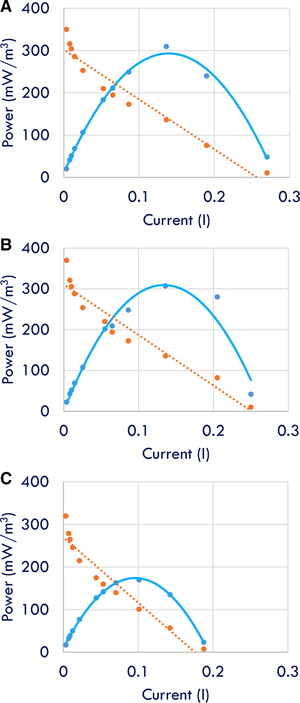
Power density profiles in microbial desalination cells affected by COD, TDS, and microalgae biomass concentrations (ABS): (A) COD, TDS, ABS – 300, 30, 0.3; (B) COD, TDS, ABS – 200, 20, 0.2; and (C) COD, TDS, ABS ‐ 100, 10, 0.1

The power density profiles are shown for different combinations of COD, TDS, and microalgae biomass concentrations in Figure 6. It can be noted that the general trend shows that the power density increases with increase in COD, TDS, and microalgae biomass concentrations. Figure 6B demonstrates that there could be a better combination of COD, TDS, and microalgae biomass concentrations as the maximum power density of over 300 mWm^−3^. However, it should be noted that the polarization curve does not represent the total power production of the bioelectrochemical system, but it shows the potential maximum power density and current production that could be achieved in a given configuration. In a broader energy perspective, wastewater treatment requires 0.5–2 kWhm^−3^ depending on the treatment scheme. However, MDCs produce bioelectricity while desalinating the saline water due to ionic migration, which may save energy requirements for water desalination which is about 2.2 kWhm^−3^. In addition, 1.8 kWhm^−3^ of bioelectricity can be generated in MDCs by treating 1 m^3^ of wastewater. Combining the energy produced and that saved by MDCs, a total 4 kWhm^−3^ of energy savings can be realized. Microalgae can be harvested to produce additional energy in the form of biofuels 10.

### 3.5 Cultivation‐independent microbial analyses for anode compartment

**Figure 7:**
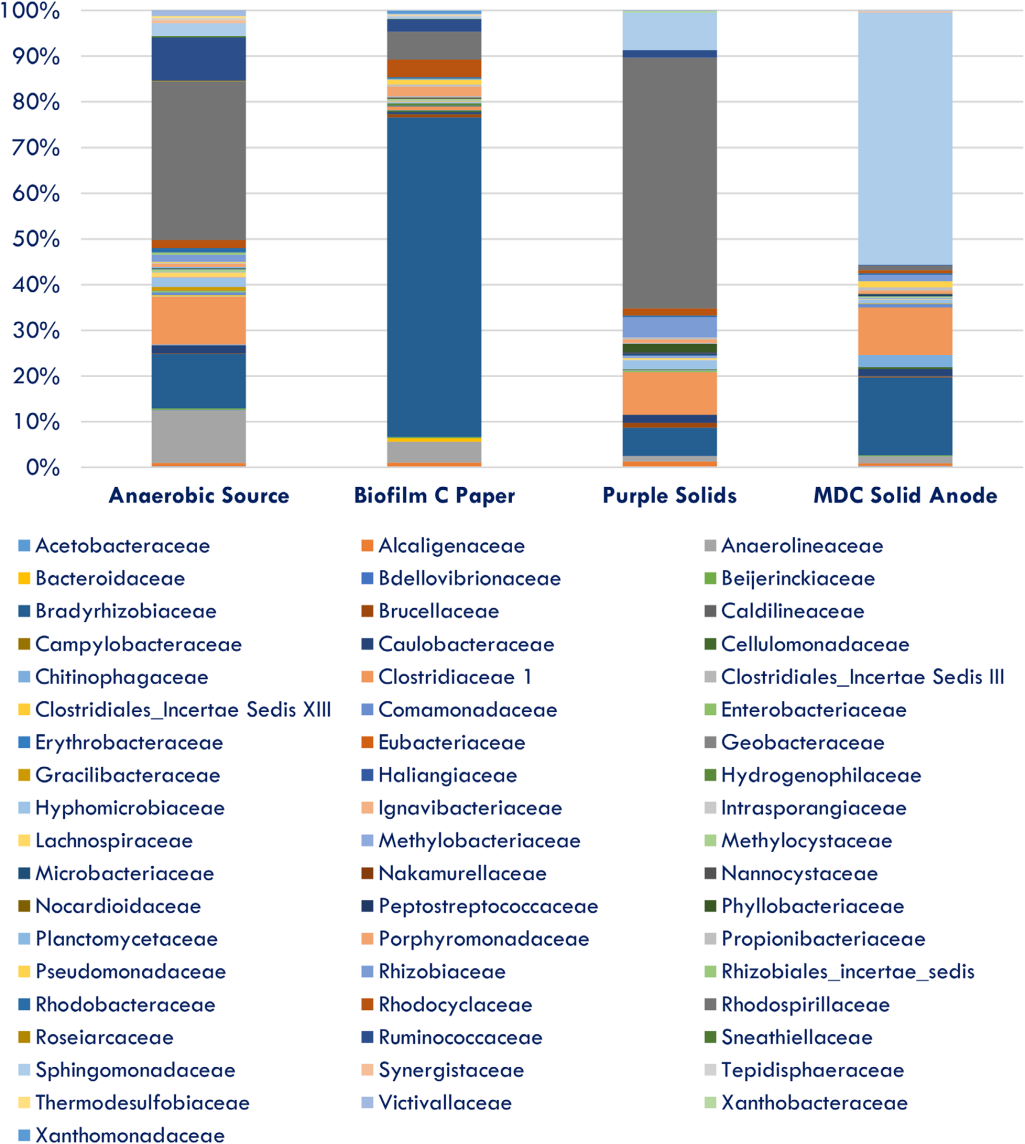
Samples classified to the family level of taxonomy using OTU classification at the 0.03 level as a percentage of total unique sequences identified

Table 4 demonstrates the direct comparison of unique OTUs classified and identified at the class level of taxonomy. Overall, each pairwise comparison demonstrated that there were unique classes within each sample type. Furthermore, taxonomical classification of unique OTUs to the family level shows that distribution of families varied by sample type, with *Azosporillium*, a key member of nitrogen fixing populations, dominating two of four sample types (data not shown). *Rhodopseudomonas*, a purple nonsulfur phototroph, was also a dominant member of three of four sample types (data not shown). Members of the *Clostridiaceae* family were also evident and indicative of an anaerobic inocula source (Figure 7). *Bradyrhizobiaceae* was a dominant member of all four sample types, particularly the carbon membrane paper, while *Hyphomicrobiaceae* contributed to the purple color from the purple solids sample. *Sphingomonadaceae* dominated the MDC solid anode and indicates a chemoheterotrophic environment.

**Table 4:** Pairwise library comparisons at the class level of taxonomy using RDP Library Compare comparison statistic


	Pairwise Library Comparison–*p*‐value					
Taxonomy–Class	Anaerobic Source/Biofilm C Paper	Anaerobic Source/Purple Solids	Biofilm C Paper/Purple Solids	MDC Solid Anode/Purple Solids	MDC Solid Anode/Biofilm C Paper	MDC Solid Anode/Anaerobic Source
“Bacteroidia”	**1.40E‐04**	7.79E‐01	**3.40E‐04**	1.00E+00	**3.40E‐04**	7.79E‐01
“Lentisphaeria”	6.54E‐02	**3.91E‐03**	2.50E‐01	5.00E‐01	6.25E‐01	**2.15E‐02**
Acidobacteria_Gp1	2.50E‐01	1.80E‐01	**1.56E‐02**	4.35E‐01	**1.95E‐03**	**3.86E‐02**
Acidobacteria_Gp16	6.88E‐01	**4.43E‐03**	**1.31E‐03**	5.62E‐01	**7.39E‐03**	**2.13E‐02**
Acidobacteria_Gp3	6.25E‐01	1.80E‐01	7.03E‐02	7.74E‐01	1.25E‐01	2.89E‐01
Acidobacteria_Gp4	6.25E‐02	6.25E‐02		5.00E‐01	5.00E‐01	2.19E‐01
Actinobacteria	**8.60E‐04**	**2.71E‐02**	2.22E‐01	**3.32E‐02**	3.58E‐01	**4.13E‐05**
Alphaproteobacteria	**6.00E‐14**	**6.00E‐14**	1.94E‐01	7.72E‐01	3.12E‐01	**6.00E‐14**
Anaerolineae	**6.34E‐05**	**6.00E‐14**	**2.67E‐05**	4.12E‐01	**3.40E‐04**	**6.00E‐13**
Bacilli	2.50E‐01	2.50E‐01		5.00E‐01	5.00E‐01	6.25E‐01
Betaproteobacteria	**2.03E‐02**	3.90E‐01	**1.58E‐03**	**1.51E‐02**	**6.68E‐08**	**1.12E‐03**
Caldilineae				5.00E‐01	5.00E‐01	5.00E‐01
Clostridia	**6.00E‐14**	**6.68E‐08**	**2.15E‐08**	1.00E+00	**2.15E‐08**	**6.68E‐08**
Cytophagia	5.00E‐01		5.00E‐01		5.00E‐01	
Deltaproteobacteria	4.53E‐01	6.25E‐01	2.19E‐01	5.00E‐01	6.25E‐02	2.50E‐01
Endomicrobia	1.25E‐01	3.75E‐01	5.00E‐01	5.00E‐01		1.25E‐01
Epsilonproteobacteria	5.00E‐01		5.00E‐01		5.00E‐01	
Gammaproteobacteria	**4.88E‐02**	7.74E‐01	**2.66E‐02**	**4.14E‐02**	8.49E‐01	7.19E‐02
Ignavibacteria	1.00E+00	5.00E‐01	5.00E‐01		5.00E‐01	5.00E‐01
Ktedonobacteria				5.00E‐01	5.00E‐01	5.00E‐01
Phycisphaerae	3.75E‐01	1.25E‐01	5.00E‐01	5.00E‐01	1.00E+00	3.75E‐01
Planctomycetia	1.00E+00	6.25E‐01	6.25E‐01	5.00E‐01	2.50E‐01	2.50E‐01
Sphingobacteria	1.00E+00	5.00E‐01	5.00E‐01	**5.96E‐08**	**8.05E‐07**	**8.05E‐07**
Synergistia	1.25E‐01	**3.13E‐02**	5.00E‐01	2.50E‐01	6.25E‐01	2.89E‐01

Highlighted/bolded comparisons were significant (*p* < 0.05).

**Figure 8:**
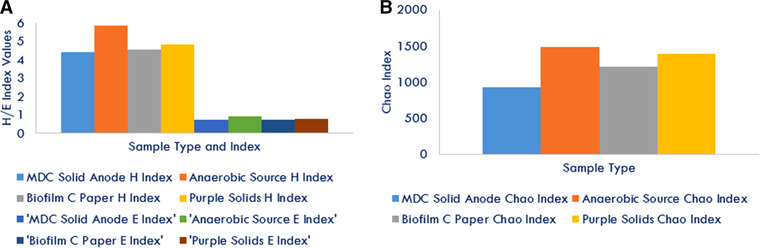
(A) Shannon H and E diversity indices by sample type based on OTU classification at the 0.03 level; (B) Species richness, chao 1 index, was estimated based on 1063 random sub sampled unique sequences from each sample type

The Shannon diversity H index estimation, based on a sub‐sampling of 1063 unique sequences from each sample type, demonstrated that three of four MDC sample locations were similar to one another, with the anaerobic source as the only difference (Figure 8). The anaerobic source was the base for the other three sample types and was the furthest differentiated from the MDC. The Shannon equitability index (E) indicated that all sample types were nearly completely even based on OTU distribution, thus also indicating one singular base source for the microbial population. The anaerobic source was the most evenly distributed of the four sample types with the other three classified at nearly 0.75 evenness (maximum of 1). Based on H’ and E’ indices, it appears that the anaerobic source was more diversified and evenly distributed than the other sample types. The species richness estimator (Chao) demonstrated that the anaerobic source was the richest in unique species, while the MDC solid sediment was the least, which corroborated and explained increased H’ index in the anaerobic source.

## 4 CONCLUDING REMARKS

This study demonstrated the optimization study of photosynthetic microbial desalination cell technology. Experimental studies elucidated the issues related to reproducibility and reliability of the process outcomes. RSM optimization method allowed for deriving relationships between the different process parameters. Understanding the interdependence and simultaneous responses of process variables in MDCs is critical for their practical applications. A maximum voltage of 0.17 V was produced from low substrate synthetic wastewater and a desalination rate of up to 30% is feasible in photosynthetic MDCs. This study proved that simultaneous energy and water recovery can be feasible from low substrate/strength wastewaters, however, long‐term, pilot‐scale studies are required for proper evaluation of the techno‐economic feasibility.

## CONFLICT OF INTEREST

The authors have declared no conflict of interest.
